# RBM15 in diseases: Molecular mechanisms and clinical opportunities from RNA m^6^A methylation

**DOI:** 10.1016/j.gendis.2025.101901

**Published:** 2025-10-28

**Authors:** Fengze Li, Junzhe Liu, Na Wang, Zhihong Zhou, Linzhen Huang, Qiankun Ji, Jingying Li

**Affiliations:** a Department of Comprehensive Intensive Care Unit, The 2nd Affiliated Hospital, Jiangxi Medical College, Nanchang University, Nanchang, Jiangxi 330006, China; b Department of Neurosurgery, The 2nd Affiliated Hospital, Jiangxi Medical College, Nanchang University, Nanchang, Jiangxi 330006, China; c Department of Neurosurgery, Zhoukou Central Hospital, Zhoukou, Henan 466000, China; d College of Queen Mary, Nanchang University, Nanchang, Jiangxi 330006, China; e Jiangxi Key Laboratory of Neurological Tumors and Cerebrovascular Diseases, Nanchang, Jiangxi 330006, China; f Jiangxi Health Commission Key Laboratory of Neurological Medicine, Nanchang, Jiangxi 330006, China; g Institute of Neuroscience, Nanchang University, Nanchang, Jiangxi 330006, China; h The MOE Basic Research and Innovation Center for the Targeted Therapeutics of Solid Tumors, The 2nd Affiliated Hospital, Jiangxi Medical College, Nanchang University, Nanchang, Jiangxi 330006, China; i Department of Cardiology, Zhoukou Central Hospital, Zhoukou, Henan 466000, China

**Keywords:** Cancer, Disease, Gene regulation, m^6^A methylation, RBM15, Therapeutic target

## Abstract

RNA m^6^A methylation is the most common type of RNA modification, and RBM15 regulates various cellular processes by writing m^6^A methylation on RNA. m^6^A methylation mediated by RBM15 significantly affects RNA stability and translational efficiency, thereby regulating gene expression. In-depth studies have revealed that RBM15 affects the progression of various diseases by regulating the expression of multiple genes, and its m^6^A methylation modification process is also considered to be an effective therapeutic target. In this paper, we review the latest research progress on the regulation of m^6^A methylation modification of RBM15, its molecular regulation in various diseases, such as cancer and metabolic diseases, and the potential therapeutic drugs derived from it, with a view to providing therapeutic strategies for the subsequent research on RBM15 and gene therapy targeting RBM15.

## Introduction

**Figure 1:**
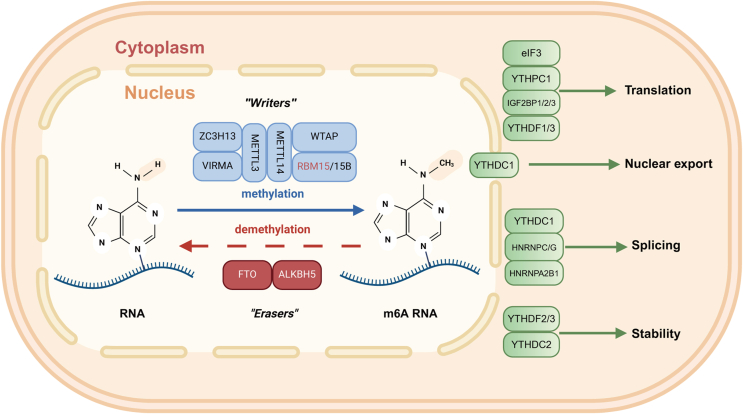
The basic mechanism of m^6^A methylation. m^6^A methylation involves the assembly of a complex by methylation transferases, notably METTL3 and METTL14, alongside other writer proteins. This complex appends a methyl (-CH3) group to the RNA molecule, a modification that can be subsequently reversed or erased by specific proteins like ALKBH5 and FTO, among others, by eliminating the m^6^A mark. Following this, the YTH domain protein family and IGFBP proteins, among others, further interact with the m^6^A-modified RNA. These reader proteins recognize the m^6^A modification and modulate various RNA functions, including translation, nuclear export, splicing, and stability, thereby exerting a regulatory influence on RNA metabolism and function.

N6-methyladenosine (m^6^A) methylation occupies an extremely important position in RNA modification. It has been considered a post-transcriptional regulatory marker found in various types of RNAs, including messenger RNAs (mRNAs), transfer RNAs (tRNAs), ribosomal RNAs (rRNAs), cyclic RNAs (circRNAs), microRNAs (miRNAs), and long-stranded non-coding RNAs (lncRNAs), altering the local secondary structure and thus affecting the interaction of RNA with RNA-binding proteins (RBPs).^1^ This interaction can be either facilitative or inhibitory, resulting in direct regulation of RBP binding to m^6^A-modified RNA.^2^ In the 5′-untranslated regions (UTR) region of mRNA, methylation modifications play important roles in mRNA splicing, editing, stability, degradation, and polyadenylation.^3^^,^^4^ While in the 3′-UTR, methylation modifications contribute to the exonuclear translocation of mRNAs, translation initiation, and the maintenance of mRNAs together with poly(A) binding protein structural stability.^5^^,^^6^ At the mechanistic level, m^6^A methylation is accomplished through an enzymatic reaction known as the m^6^A methyltransferase complex (MTC). This complex consists of several proteins, including the core enzymes methyltransferase-like 3 (METTL3) and METTL14, and cofactors such as Wilms' tumor 1-associating protein (WTAP) and RNA-binding motif protein 15 (RBM15).^7^, ^8^, ^9^ These enzymes and factors act synergistically to transfer methyl groups from S-adenosylmethionine (SAM) to specific sites in the RNA, forming m^6^A modifications (Fig. 1).^10^ This process thereby regulates several biological processes in cells, such as tissue development, stem cell self-renewal, differentiation, heat shock, and DNA damage response.^11^, ^12^, ^13^, ^14^, ^15^

RBM15, initially discovered by Wiellette et al in 1999 and named by Ma et al, has been extensively studied.^16^^,^^17^ Research indicates it belongs to the split ends (SPEN) family and can also be regarded as a part of the RBM protein family.^18^ As an RNA methyltransferase, RBM15 significantly impacts tumor progression. The physiological functions of RBM15 in cells have been well reviewed by Yuan et al.^19^ In pathological tissues, RBM15 modulates the transcriptional activity of downstream target genes via m^6^A modification. This epigenetic regulation influences critical biological processes, including cell proliferation, apoptosis, invasion, and migration.^20^

In recent years, a substantial body of research has delineated a pivotal role of RBM15, an m^6^A-binding protein, in the neoplastic transformation of cancer cells. This protein exerts its influence by modulating the expression of a plethora of genes implicated in oncogenic processes.^21^ Here, we review recent advances in the molecular regulatory mechanisms of RBM15 in various diseases and the potential therapeutic drugs derived from these.

## Overview of RBM15

### The structure of RBM15

**Figure S1:**
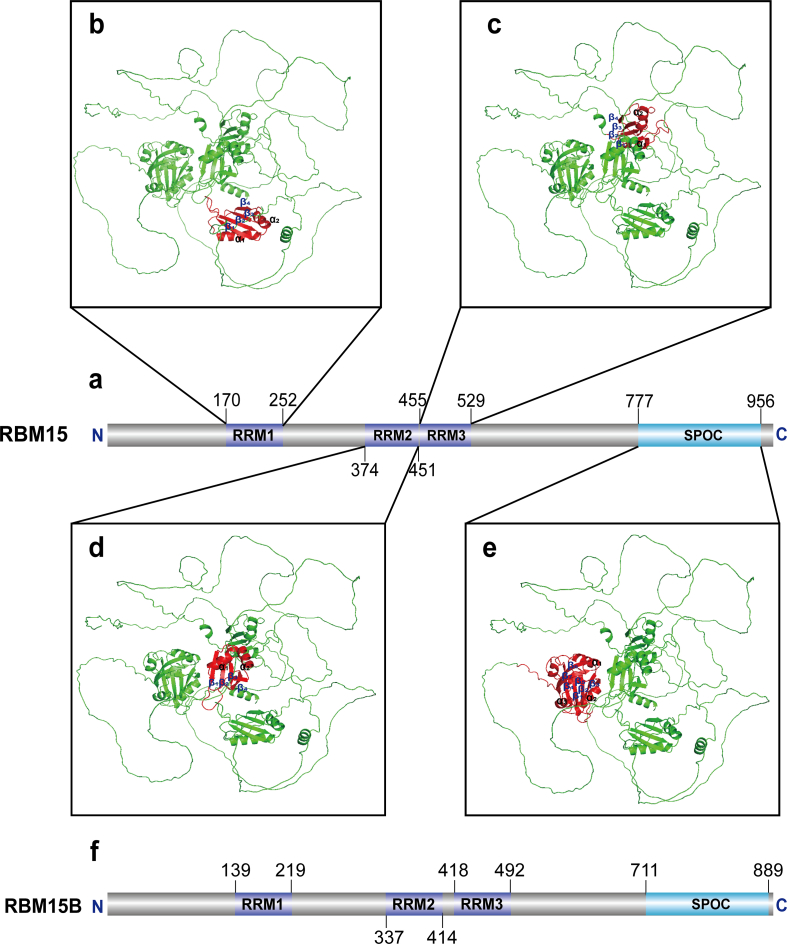
Structural model of the RBM15/15B. i) Schematic representation of the structural domains of RBM15 protein: RRM1 (aa 170–252), RRM2 (aa 374–451), RRM3 (aa 455–529), and SPOC (aa 777–956). ii) RBM15 RRM1 protein structure model: composed of 2 α helices and 4 β sheets. iii) RBM15 RRM2 protein structure model: composed of 2 α helices and 4 β sheets. iv) RBM15 RRM3 protein structure model: composed of 2 α helices and 4 β sheets. v) RBM15 SPOC protein structure model: composed of 2 α helices and 4 β sheets. vi) Schematic representation of the structural domains of the RBM15B protein: RRM1 (aa 139–319), RRM2 (aa 337–414), RRM3 (aa 418–492), and SPOC (aa 711–889).

Three N-terminal RNA recognition motifs (RRMs) and one C-terminal spen paralog and ortholog C-terminal (SPOC) structural domain are the major protein structural domains of RBM15 (Fig. S1). A common RRM is approximately 90 amino acids long with a typical β1α1β2β3α2β4 topology, forming a four-stranded β-sheet packed against two α-helices.^22^ RRMs are the approach of binding RBPs to sequence-specific motifs or RNA secondary structures, or a combination of both, with low or high affinity and specificity.^23^ Research has consistently demonstrated that a subset of RBM proteins utilize two external β-strands for binding, whereas others engage loops or the C- and N-terminal domains to achieve enhanced affinity.^24^ These RRMs are intricately linked with a myriad of post-transcriptional regulatory processes, playing a pivotal role in the modulation of gene expression.^25^, ^26^, ^27^ The SPOC structural domain is a 15–20 kDa protein structural domain forming a twisted β-barrel structure, which comprises seven β-strands and a variable number of α-helices.^28^, ^29^, ^30^ SPOC structural domains are at the core of protein–protein interactions, typically in a phosphorylated serine-dependent manner.^31^ As such, RBM15 SPOC has been shown to bind to the unstructured LPDSD motif of histone H3 lysine 4 (histone H3K4me3) methyltransferase and also to the LSETD motif of WTAP.^31^^,^^32^ This helps explain why the abundance of m^6^A methylation in cells is strongly correlated with the presence of RBM15 and its SPOC structural domains.

### RBM15 and RBM15B

Remarkably, RBM15 and its homologous counterpart RBM15B exhibit considerable structural and sequence similarities in their structural domains. Both have been documented to play pivotal post-transcriptional roles in RNA modification, splicing, and translocation processes.^33^, ^34^, ^35^ The presence of three RRMs and a SPOC structural domain in both proteins indicates their potential to function as catalysts for m^6^A methylation. A research study elaborates that METTL3, in a manner reliant on WTAP, is capable of associating with RBM15/15B to constitute an MTC complex.^36^

Given the sequence and structural similarities between RBM15 and RBM15B, their homology provides a foundation for redundant functions. A notable example is the RBM15/15B-mediated silencing of X-inactive specific transcript (XIST) transcription. However, the simultaneous knockdown of both RBM15 and RBM15B results in a significant suppression of XIST-mediated gene silencing, such as that of glypican 4 (Gpc4).^36^ Conversely, RBM15B does not appear to compensate for or replace the functions of RBM15 when its expression is diminished or absent during mouse embryonic development.^34^

In addition to their function as transcriptional regulators, RBM15/15B also serve as cofactors that facilitate the nuclear export of mRNA. RBM15 has been demonstrated to interact with nuclear RNA export factor 1 (NXF1), augmenting the export and expression of mRNA harboring the RNA transport element (RTE).^37^ RBM15B also influences NXF1's translocation function, particularly through the activation of RTE-containing reporter gene RNAs.^34^ This effect is predominantly reliant on RBM15, highlighting the pivotal role of RBM15 in the process. RTE, identified in mouse brain pool A granules, is crucial not only for the migratory attributes of these granules but also for its role in the regulatory mechanisms of RNA export,^37^, ^38^, ^39^ as evidenced by its ability to disrupt the Rev-RRE mechanism in HIV-1.^39^ Although RTE does not exhibit high binding affinity for NXF1, the connection between NXF1 and RTE is likely facilitated through RBM15 and RBM15B.^40^ Specifically, NXF1 interacts with the C-terminal region of RBM15/15B via its nuclear transport factor 2 (NTF2)-like domain. Intriguingly, RBM15B presents fewer interaction sites with NXF1 compared with RBM15, prompting an exploration of the functional redundancy between these two proteins.^41^^,^^42^

### The functions of RBM15

**Figure 2:**
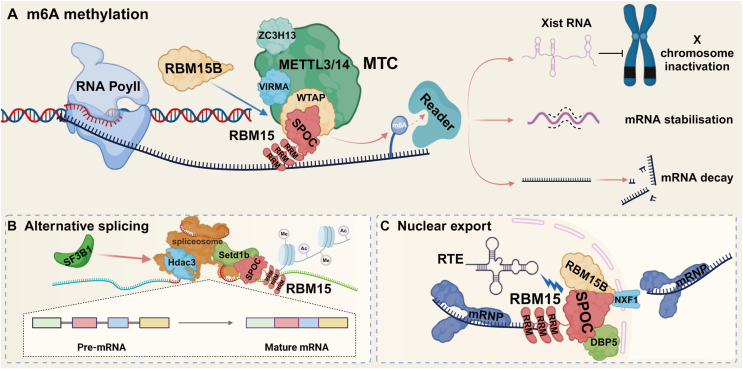
Cellular mechanisms involved in RBM15. i) RBM15 directs the METTL3–METTL14 complex to target RNAs bound to the RRM structural domain through binding to RBM15B, using its SPOC structural domain, mediated by WTAP. This process promotes m^6^A modification on the RNA, which subsequently triggers a variety of biological responses, including, but not limited to, silencing of the X chromosome and enhancement or attenuation of mRNA stability. ii) As a component of the spliceosome complex, RBM15 is able to interact with molecules such as histone deacetylase 3 (Hdac3) and histone methyltransferase (Setd1b) to fine-tune the selective splicing process of target RNAs using epigenetic regulatory mechanisms such as H4 acetylation and H3k4me3. In addition, RBM15 further influences the splicing patterns of specific genes by collaborating with key splicing factors such as SF3B1 to regulate gene expression. iii) RBM15 also plays the role of a nuclear export factor, and its SPOC terminus interacts with proteins such as DBP5 and NXF1, which together promote the nuclear export process of the mRNA-ribonucleoprotein (mRNP) complex. In addition, by interacting with RBM15B, RBM15 can activate the function of RNA transporter element (RTE) in the nucleus, which further promotes the transport and localization of RNA in the cell.

RBM15, a component of the multi-protein complex MTC, collaborates with other proteins, such as METTL3, METTL14, and WTAP, to catalyze the methylation at the N6 position of adenosine in RNA molecules.^43^ In this complex, RBM15 may help to localize the active center of the methyltransferase by interacting with other components, thus precisely regulating the selection of methylation sites.^36^ Furthermore, RBM15 plays a pivotal role in the regulation of diverse biological processes. It is crucial for the Xist-mediated inactivation of X chromosomes, a process that ensures dosage compensation in cells with different sex chromosome complements.^44^ Additionally, RBM15 has the potential to modulate alternative splicing of c-Mpl RNA through epigenetic mechanisms, which can influence the functionality of the resulting protein isoforms.^45^^,^^46^ Moreover, RBM15 facilitates mRNA transport from the nucleus to the cytoplasm, a process that is essential for gene expression regulation and can occur through multiple pathways.^36^ These functions make RBM15 play an indispensable role in cell growth, differentiation, and functional maintenance (Fig. 2). Notably, RBM15 is essential for the development of organic tissues, particularly in sustaining long-term hematopoietic stem cell homeostasis, as well as in the differentiation of megakaryocytes and B cells.^47^^,^^48^

### The interaction of RBM15 with readers

Comprehensive analysis concludes that the pathogenic mechanisms of RBM15 in various diseases are predominantly orchestrated through the m^6^A modification pathway. Upon the introduction of m^6^A modifications by RBM15 on RNA molecules, these specific sites are recognized and engaged by dedicated reader proteins. The diversity of reader proteins exerts distinct effects on m^6^A-marked mRNAs: some stabilize the mRNA by binding to these modifications, such as the insulin-like growth factor 2 mRNA-binding protein (IGF2BP) family, thereby protecting them from degradation, while others may accelerate the decay process or alter translational efficiency. This observation elucidates the varied fates and functional outcomes of RBM15-modified mRNAs in disease contexts. Reader proteins that have been validated to recognize RBM15-modified mRNAs are summarized in Table 1. Among them, the very different regulation of mRNA fate by YTH domain family 1 (YTHDF1) and YTHDF2 is noteworthy, although the YTH structural domains that play a central role are almost identical in both.^49^ Mechanistically, the RBM15/METTL3 complex enhances m^6^A modification and promotes translation of downstream proteins by interacting with the 359A site of the YTHDF1 protein 50. In contrast, YTHDF2 binds, in most cases, to the 3′-UTR region of the mRNA modified by the complex and accelerates the degradation of RBM15-modified mRNAs by recruiting the carbon catabolite repression 4 (CCR4)-negative on TATA-less (NOT) complex, which reduces the expression of the corresponding proteins.^51^

**Table 1:** The interaction between RBM15 and the “reader” protein.

Disease	Reader	mRNA	Effect
Laryngeal cancer	IGF2BP3	KDM5B	Stabilise
NSCLC	IGF2BP3	CBR3-AS1	Stabilise
LSCC	IGF2BP3	TMBIM6	Stabilise
HCC	IGF2BP3	VEGFA	Stabilise
HCC	IGF2BP1	YES1	Stabilise
HCC	YTHDF2	VEGFA	Stabilise
HCC	YTHDF2	cFAM210A	Decay
SCI	YTHDF2	SOX18	Decay
PE	YTHDF2	CD82	Decay
BLCA	YTHDF1	ENO1	Stabilise

Abbreviations: NSCLC, Non-Small cell lung cancer; LSCC, Lung squamous cell cancer; HCC, hepatocellular carcinoma; PE, Pre-eclampsia; SIC, Sepsis-induced cardiomyopathy; BLCA, bladder cancer; IGF2BP3; Insulin Like Growth Factor 2 mRNA Binding Protein 3; YTHDF2, YTH N6-Methyladenosine RNA Binding Protein F2; IGF2BP1, Insulin-Like Growth Factor 2 mRNA Binding Protein 1; YTHDF1,YTH N6-Methyladenosine RNA Binding Protein F2; KDM5B, Lysine Demethylase 5B; TMBIM6, Transmembrane BAX Inhibitor Motif Containing 6; VEGFA, Vascular Endothelial Growth Factor A; YES1, YES Proto-Oncogene 1, Src Family Tyrosine Kinase; SOX18, SRY-Box Transcription Factor 18; ENO1, Enolase 1.

## The role of RBM15 in disease

The key role of RBM15 as an m^6^A methylase is reflected in its participation in the m^6^A methylation process of RNA as a component of MTC and its ability to maintain normal cellular functions by regulating a variety of biological processes. Meanwhile, any dysregulation in the expression or functionality of RBM15 may be intricately associated with the etiology and progression of various diseases, thereby rendering it a pivotal target for both disease research and therapeutic intervention (Table 2).^52^

**Table 2:** Roles of RBM15 in human disease.

Role	Disease	Upstream	Target	Mechanism	Function	Ref.
Supporter	Laryngeal cancer	–	KDM58	Stabilise KDM58 mRNA	• Cell proliferation • Rrug resistance • Ferroptosis	65
	LSCC	–	TMBIM6	Stabilise TMBIM6 mRNA	• Cell proliferation • Migration • Invasion	64
	LUAD	*–*	RASSF8	Stabilise RASSF8 mRNA	• Cell proliferation • Migration • Invasion	69
	HCC	–	VEGFA	Stabilise VEGFA mRNA	• Cell growth • Invasion • Angiogenesis	55
	HCC	–	YES1	Stabilise YES1 mRNA	• Cell proliferation • Invasion	91
	HCC	HBx	cFAM210A	Decay cFAM210A mRNA	• Cell proliferation • Malignant transformation	93
	LC	–	TGF-β/Smad2	Stabilise TGF-β/Smad2 related mRNA	• Cell proliferation • Migration	68
	OS	–	circ-CTNNB1	Stabilise circ-CTNNB1 mRNA	• Cell proliferation • Migration • Glycolysis	77
	OC	TGF-β	MDR1	Stabilise MDR1 mRNA	• Cell proliferation • Drug resistance	129
	CC	–	OTUB2	Stabilise OTUB2 mRNA	• Cell proliferation • Migration	79
	CC	–	JAK-STAT	Stabilise JAK-STAT related mRNA	• Cell proliferation • Migration • Invasion	80
	CC	HPV E6	c-myc	Stabilise c-myc mRNA	• Cell proliferation	84
	CC	–	DCN	Stabilise DCN mRNA	• Malignant transformation • Proliferation • Migration	85
	CC	–	HEIH	Stabilise HEIH mRNA	• Cell proliferation • Migration • Cell dryness	86
	CRC	–	KLF1	Stabilise KLF1 mRNA	• Cell proliferation • Migration	100
	CRC	–	MyD88	Stabilise MyD88 mRNA	• Cell proliferation • Migration • Apoptosis	99
	BLCA	TGF-β/Smad2/	ENO1	Stabilise ENO1 mRNA	• Cell proliferation	53
	BLCA	–	lncRNAs	Stabilise lncRNAs	• Cell proliferation • Invasion • Migration	127
	ccRCC	EP300/CBP	CXCL11	Stabilise CXCL11 mRNA	• Cell proliferation • Migration • Invasion • EMT	124
	CCRCC	–	CCNB1	Stabilise CCNB1 mRNA	• Cell proliferation • Migration	169
	ESCA	–	miR-3605-5p	Stabilise miR-3605-5p	• Cell proliferation • Migration	131
	KSHV	ORF57	ORF59	Stabilise ORF59 mRNA	• Tumor progression	141
	GBM	miR-184-3p	DLG3	Stabilise DLG3 mRNA	• PMT • Radioresistance	134
	BC	–	SSP	Stabilise SSP mRNA	• Cell proliferation • Migration • Invasion	111
	Diabetes	–	CLDN4	Stabilise CLDN4 mRNA	• Cell insulin sensitivity	150
	DN	–	AGE-RAGE	Stabilise AGE-RAGE related mRNA	• Cell proliferation • Inflammation • Oxidative stress • Pyrosis	152
	MI	–	NAE1	Stabilise NAE1 mRNA	• Apoptosis	144
	AD	–	–	Mediate m6A methylation	• Cell polarization • Glycolysis	164
	AAA	–	CASP3	Stabilise CASP3 mRNA	• Apoptosis	157
Suppressor	PCa	–	R-loop	Stabilise R-loop	• Cell proliferation • Migration	120
	PCa	–	p53 target mRNA	Stabilise p53 target mRNA	• Cell proliferation • Apoptosis	116
	PCa	UBA1	TPM1	Decay TPM1 mRNA	• Cell proliferation • Migration	136
	THCA	–	–	Mediate m6A methylation	• Cell proliferation • Migration • Invasion	118
	AMKL	–	Setd1b	Enhances the pathogenicity of RBM15-Mkl1	• Cell proliferation • Growth	22
	NAFLD	–	RNF5	Stabilise RNF5 mRNA	• Cellular inflammation • Oxidative stress	154

Abbreviations: LSCC, Lung squamous cell cancer; LUAD, lung adenocarcinoma; HCC, hepatocellular carcinoma; LC, lung cancer; OS, osteosarcoma; OC, ovarian cancer; CC, cervical cancer; CRC, colorectal cancer; BLCA, bladder cancer; ccRcc, clear cell renal cell carcinoma; ESCA, oesophagal carcinoma; KSHV, Kaposi's sarcoma-associated herpesvirus; GBM, glioblastoma; BC, breast cancer; DN, diabetic nephropathy; MI, myocardial infarction; AD, aortic aneurysm and dissection; AAA, abdominal aortic aneurysm; PCa, prostatic cancer; THCA, thyroid carcinoma; AMKL, acute megakaryocytic leukaemia; NAFLD, non-alcoholic fatty liver disease; PMT:proneural–mesenchymal transition; EMT: epithelial–mesenchymal transition.

### RBM15 as a cancer supporter

#### Leukemia

RBM15 plays an integral role in hematopoiesis, as evidenced by its N-terminal capacity to interact with RBPJκ, thereby activating the Notch signaling pathway and triggering apoptosis in myeloid hematopoietic cells.^53^ This interaction is crucial for the regulation of hematopoietic cell fate and survival, underscoring the significance of RBM15 in the maintenance of hematopoietic homeostasis.^48^^,^^53^ Therefore, RBM15 is often aberrantly expressed in leukemia. In chronic myeloid leukemia, the expression of RBM15 is notably elevated. Studies have demonstrated that the down-regulation of RBM15 arrests cell cycle progression, thereby inducing apoptosis. Moreover, a potential correlation has been revealed between RBM15 and the Notch signaling pathway, which may have significant implications for chronic myeloid leukemia pathogenesis and treatment.^54^ Acute megakaryoblastic leukemia is a subtype of acute myeloid leukemia characterized, among other things, by the presence of the fusion gene RBM15-megakaryoblastic leukemia 1 (MKL1), resulting from the chromosomal translocation t (1; 22) (p13; q13).^16^^,^^55^ The RBM15-MKL1 fusion protein is overexpressed in acute megakaryoblastic leukemia cells. It is the only known recurrent mutation involving the m^6^A writing protein complex. Mechanistic insights reveal that the RBM15-MKL1 fusion protein modulates cellular proliferation through interactions with the H3K4me3 methyltransferase-SET domain containing 1b (Setd1b). This interaction occurs via the SPOC domain, thereby influencing the enzymatic activity of Setd1b and consequently, the proliferation of cells.^32^

#### Head and neck squamous cell carcinoma

While head and neck squamous cell carcinoma (HNSCC) treatment has improved considerably over the past few decades, the 5-year overall survival of patients remains dismal, and there is a need to identify viable molecular markers and associated regulatory mechanisms.^56^^,^^57^ Research has shown prominent expression of RBM15 in HNSCC, with the insulin-like growth factor 2 mRNA-binding protein (IGF2BP) family emerging as a key player in the development of this malignancy.^58^ In a study focusing on laryngeal squamous cell carcinoma, a subset of HNSCC, RBM15 was identified as the most significantly differentially expressed m^6^A methyltransferase among the “writer” proteins.^59^ Subsequent investigations have revealed that IGF2BP3 modulates the m^6^A modification of transmembrane BAX inhibitor motif containing 6 (TMBIM6) mRNA by RBM15. This regulation occurs through the binding of IGF2BP3 to the m^6^A site within the 3′-UTR of TMBIM6, thereby affecting the stability of TMBIM6 mRNA and consequently promoting cancer cell proliferation, migration, and invasion.^60^ In the context of laryngeal cancer, a distinct subtype within the broader category of HNSCC, the investigators additionally discovered that after the m^6^A methylation modification of RBM15 and the response mediated by insulin-like growth factor binding protein 3 (IGFBP3), lysine demethylase 5B (KDM5B) functions to hinder ferroptosis by suppressing the expression of fike family member 4 (FER1L4) and enhancing the levels of glutathione peroxidase 4 (GPX4). This mechanism ultimately fosters cellular proliferation.^61^ However, other specific molecular mechanisms involved in RBM15 in HNSCC remain unknown.

#### Lung cancer

Lung cancer is the leading cause of death from malignant tumors worldwide.^62^ Non-small cell lung cancer is the most common type of lung cancer, with the most common subtype being lung adenocarcinoma.^63^ The researchers initially recognized an up-regulation of RBM15 in lung cancer cells. Following this observation, they found that when RBM15 was silenced, there was an elevation in the levels of ferrous iron (Fe^2+^) and the labile iron pool, while the expression of transforming growth factor-β (TGF-β) and SMAD family member 2 (Smad2) decreased. This indicates that RBM15 serves to suppress iron metabolism and facilitates tumor growth, migration, and invasion.^64^ A recent study elucidated the pathogenesis of RBM15 in lung adenocarcinoma: RBM15 enhances stability and promotes cell invasion and migration by writing inhibitory methylation modifications to Ras-association domain family member 8 (RASSF8) mRNA.^65^ In addition, other studies have shown that the interaction of nucleoside diphosphate kinase 6 (NME6), SET domain containing 2 (STED2), and RBM15 fosters lung adenocarcinoma progression and correlates with an unfavorable prognosis.^66^^,^^67^

#### Osteosarcoma

Osteosarcoma is the most prevalent primary bone cancer and the most common malignancy in adolescents.^68^ Within osteosarcoma cell lines and tissues, the homeostasis of m^6^A and its regulatory machinery is often perturbed, notably implicating RBM15 as a potential prognostic biomarker linked to m^6^A.^69^, ^70^, ^71^ Specifically, RBM15 has been shown to be a marker exclusive to metastatic osteosarcoma, where it enhances cellular invasion, migration, and metastasis, as evidenced by functional assays and animal models.^72^ At the molecular level, the interaction between circular RNA catenin beta 1 (circ-CTNNB1) and RBM15, mediated by the RRM domain, is crucial for the m^6^A methylation of glycolytic enzymes: hexokinase-2 (HK2), glucose-6-phosphate isomerase (GPI), and phosphoglycerate kinase-1 (PGK1). This interaction is instrumental in promoting aerobic glycolysis and subsequent progression of osteosarcoma in affected cells.^73^

#### Cervical cancer

Cervical cancer (CC) is the predominant gynecological malignancy globally, with cervical squamous cell carcinoma responsible for nearly 75% of CC-related mortalities.^74^ RBM15 has been identified as significantly overexpressed in CC and implicated in the modulation of multiple intracellular signaling pathways.^75^ Specifically, RBM15 has been shown to modulate the m^6^A methylation of OTU dejubiquitinase, ubiquitin aldehyde binding 2 (OTUB2), thereby activating the AKT/mTOR pathway, which enhances CC cell proliferation and migration and inhibits apoptosis.^75^ Furthermore, RBM15 down-regulation in CC cells was observed to significantly reduce the expression of proteins associated with the JAK–STAT pathway, suggesting a prominent role for RBM15 in the regulation of this pathway, which is critical for CC pathogenesis.^76^^,^^77^ The relationship between CC and human papillomavirus (HPV) is also of significant interest, as persistent infection with high-risk HPV types is a major etiological factor for CC and its precursor lesions.^78^^,^^79^ In HPV-positive CC cell lines, HPV-E6 up-regulates RBM15 expression by inhibiting ubiquitination, leading to increased binding of RBM15 to c-myc mRNA, promoting its m^6^A modification and expression. The subsequent elevation of c-myc protein contributes to cellular malignant transformation and accelerates the onset and progression of CC.^80^ Additionally, RBM15 knockdown has been reported to diminish CC tumorigenesis by mediating m^6^A modification of decorin (DCN) mRNA within CC cells, and it also impacts tumor cell proliferation, metastasis, and stemness through the stabilization of hepatocellular carcinoma up-regulated EZH2-associated (HEIH) lncRNA expression.^81^^,^^82^

#### Hepatocellular carcinoma

Hepatocellular carcinoma is one of the most common tumors of the digestive system, which has high recurrence and metastasis rates, and its patients have a poorer prognosis, especially in advanced stages.^83^^,^^84^ Analysis of The Cancer Genome Atlas (TCGA) data indicates that RBM15 is frequently overexpressed in hepatocellular carcinoma and potentially serves as an independent prognostic biomarker for overall and disease-free survival in hepatocellular carcinoma patients.^85^ RBM15 expression correlates significantly with TNM staging and metastatic potential, rendering it a valuable prognostic indicator for early-stage, hepatitis B virus (HBV)-associated hepatocellular carcinoma.^86^ Mechanistically, RBM15 facilitates the advancement of hepatocellular carcinoma via diverse signaling pathways that engage various readers. This includes methylation processes of genes like YES proto-oncogene 1 (YES1), which can be facilitated by the reliance on IGF2BP1, the methylation of vascular endothelial growth factor A (VEGFA) modulated by YTHDF2 and IGF2BP3, and the methylation of cFAM210A (a circular RNA derived from the third exon of transcript NM_001098801 of the family with sequence similarity 210, member A (FAM210A) gene) mediated by YTHDF2 dependence. Ultimately, these pathways collaborate to exacerbate the disease phenotype by stimulating cellular proliferation/invasion and angiogenesis and promoting stemness and malignant transformation.^87^, ^88^, ^89^

#### Colorectal cancer

Colorectal cancer has a high incidence and mortality rate worldwide and is a malignant tumor of the colon or rectum.^90^^,^^91^ Colorectal cancer liver metastasis is the most common cause of death from colorectal cancer.^92^ Mechanistically, RBM15 promotes both the stability and expression of KLF transcription factor 1 (KLF1) mRNA through IGF2BP3-dependent m^6^A modification, which promotes KLF1 enrichment on the SIN3 transcription regulator family member A (SIN3A) promoter and activates SIN3A transcription and also mediates the m^6^A methylation modification of MYD88 innate immune signal transduction adaptor (MyD88) mRNA in colorectal cancer cells, which ultimately contributes to the tumorigenesis of colorectal cancer liver metastasis.^93^^,^^94^

#### Gastric cancer

Gastric cancer is a prevalent malignancy of the gastrointestinal tract with a poor prognosis and is the second leading cause of cancer-related deaths worldwide.^95^ RBM15 is aberrantly overexpressed in gastric cancer, and its expression correlates with the pathological stage of gastric cancer.^96^^,^^97^ Latest studies have shown that RBM15 drives adipogenesis and promotes gastric cancer progression by regulating m^6^A modification-mediated activation of ATP citrate lyase (ACLY) in an IGF2BP2-dependent manner.^98^

#### Breast cancer

Breast cancer is the most prominent cancer in women worldwide.^99^ Basal-like breast cancer is a subtype that is associated with a poor prognosis, high risk of recurrence, and no available effective treatment strategies.^100^ RBM15 is not significantly expressed in breast cancer, but in the basal-like subtype of breast cancer, RBM15 has a particularly high frequency of copy number gain events and may serve as an important predictor to delineate the basal-like subtype from other subtypes.^101^ A recent study explains this phenomenon: RBM15 promotes cancer progression by increasing its expression through methylation of the serine synthesis pathway (SSP) mRNA, which ultimately affects the serine and glycine synthesis function of cells.^102^ Additional research has demonstrated that BARX homeobox 2 (BARX2) facilitates the advancement of breast cancer through its regulation of RBM15, while an elevated expression of msh homeobox 2 (Msx2) leads to a reduction in RBM15 levels, thereby triggering apoptosis in breast cancer cells.^103^^,^^104^

#### Thyroid cancer and prostate cancer

Thyroid cancer and prostate cancer are two of the few cancers in which RBM15 is down-regulated.^105^^,^^106^ Of particular interest is the evidence implicating RBM15 in the promotion of the above cancer progression across various studies. This body of research suggests that RBM15, along with its associated pathways, plays a nuanced and intricate role in the regulatory mechanisms within biological systems. In thyroid cancer, diminished RBM15 expression has been correlated with reduced levels of cyclin B1, cyclin D1, N-cadherin, and Vimentin, alongside an increase in p16 and phospho-cell division cycle protein 2 (p-CDC2) expression.^107^ Furthermore, the suppression of RBM15 not only deactivates the PI3K/AKT/mTOR signaling pathway but may also interact with the Notch and Wnt signaling pathways, underscoring its multifaceted role in cancer progression.^108^ In the context of prostate cancer, RBM15 orchestrates intracellular R-loop methylation, a process that implicates the regulation of IGF2BPs by R-loop m^6^A. This mechanism leads to the repression of semaphorin 3F (SEMA3F), an oncogene in prostate cancer cells, thereby highlighting RBM15's tumor-suppressive function, which we later reviewed.^109^

#### Renal cancer

Renal cancer causes 65,340 new cases and 16,970 deaths in the latest US cancer statistics report.^110^ Accounting for about 70%–80% of all kidney cancers, clear cell renal cell carcinoma is the most common primary renal cell carcinoma.^111^ The prognostic relevance of RBM15 has been independently established in clear cell renal cell carcinoma, emphasizing its significance.^112^ Researchers have illuminated a pathway in which RBM15 overexpression occurs due to histone acetylation facilitated by enhancer protein (EP300/CBP), subsequently fostering clear cell renal cell carcinoma advancement in an m^6^A-mediated fashion.^113^ Separately, studies indicate that resveratrol can modulate cancer progression by targeting RBM15's influence on cyclin B1 (CCNB1) mRNA stability and inhibiting EP300/CBP expression, thereby triggering a cellular senescence response.^114^

#### Other cancers

RBM15 is significantly up-regulated in bladder cancer. Its expression level is closely associated with immune infiltration and prognosis in bladder cancer, especially in advanced clinical stages.^115^ The RBM15/METTL3 complex was found to target not only 11 intracellular long non-coding RNAs (lncRNAs) but also enolase 1 (ENO1) via the 359A site of YTHDF2, which is activated by the TGF-β/Smad2/3 pathway, suggesting that the RBM15/METTL3 complex is inextricably linked to bladder cancer progression.^50^^,^^116^ RBM15 expression is up-regulated in ovarian cancer cells compared with normal cells, according to data from the Gene Expression Omnibus (GEO) database.^117^ In ovarian cancer cells, RBM15 mediates the methylation of multidrug resistance 1 (MDR1) and promotes the translation and expression of its mRNA at the protein level, whereas the activation of the TGF-β pathway inhibits RBM15 expression.^118^ In pancreatic cancer, elevated expression levels of RBM15 have been associated with enhanced proliferation in pancreatic cancer cells. Enrichment analysis indicates that RBM15 regulates a spectrum of cancer-related signaling pathways, notably the T-cell receptor signaling pathway, which plays a pivotal role in modulating the cell cycle and cell division of pancreatic cancer.^21^ In esophageal cancer, the expression levels of key genes within 24 major m^6^A-associated modules have been scrutinized, revealing a significant overexpression of RBM15.^119^ This regulator of m^6^A modification has been shown to modulate the expression of miR-3605-5p. Subsequent overexpression of miR-3605-5p leads to a reduction in keratin 4 (KRT4) protein levels. Consequently, the elevated expression of RBM15 enhances cell viability and migration in esophageal squamous cell carcinoma through the regulation of KRT4.^120^

### RBM15 as a cancer suppressor

#### Glioma

Gliomas form the major part of primary intracranial tumors and account for up to 81% of all malignant brain tumors.^121^ Although the overall incidence of these tumors is not considered high, they are strongly associated with a significant risk of mortality and high morbidity.^122^ In glioma, RBM15 exhibits heightened expression in both glioblastoma and brain lower-grade glioma. Studies have primarily focused on the tumor-suppressive role of RBM15. The most representative example is glioma stem cells. Glioma stem cells exhibit elevated levels of miR-184-3p, which is linked to the dysregulation of m^6^A modification by RBM15. The suppression of RBM15 leads to a decrease in m^6^A modification of discs large MAGUK scaffold protein 3 (DLG3) mRNA, a change that is instrumental in triggering interneuronal metaplasia in glioma stem cells. This process is mediated through the activation of the signal transducer and activator of transcription 3 (STAT3) pathway, which is known to promote oncogenic processes and is a key player in glioma progression.^21^^,^^123^^,^^124^

#### Prostate cancer

RBM15 also appears to play an inhibitory role in prostate cancer. RBM15 is regulated by both AZGP1 pseudogene 2 (AZGP1P2)-mediated stemness and apoptosis of prostate cancer stem cells, and mediates the decay of target tropomyosin 1 (TPM1) mRNAs, which can also be combined with FTO-IT1 to reduce the m^6^A stability of p53, leading to reduced m^6^A level and inhibiting prostate cancer progression.^105^^,^^125^ Although RBM15 displays a dual role in prostate cancer progression, experimental data indicate that overexpression of RBM15 significantly inhibits cell migration and proliferative capacity both *in vitro* and *in vivo*, suggesting that its primary function tends to be tumor suppression.^109^

#### Other cancers

The multifaceted nature of RBM15's function in endometrial adenocarcinoma underscores its significance as a standalone prognostic indicator, alongside FTO and YTHDF1. Elucidation of functional enrichment and pathway analysis networks reveals that RBM15 engages in the regulation of connective tissue development, a pivotal process for the survival prospects of endometrial adenocarcinoma patients. Additionally, a decline in RBM15 expression mirrors a reduced FTO expression in endometrial adenocarcinoma, both of which collaboratively facilitate endometrial adenocarcinoma progression.^126^ The open reading frame 57 (ORF57) protein, specifically encoded by Kaposi sarcoma-associated herpesvirus (KSHV), serves as a potent post-transcriptional regulator whose primary functions include bolstering RNA stability, facilitating the splicing of RNA, and stimulating the translation of proteins, among a broad spectrum of activities it orchestrates.^127^, ^128^, ^129^ In KSHV, RBM15 primarily promotes the accumulation and polyadenylation of ORF59 RNA in the nucleus, whereas ORF57 antagonizes RBM15 and promotes tumor progression by directly interacting with RBM15 and ORF59.^130^^,^^131^

### RBM15 in cardioprotection

Myocardial infarction, a serious condition in the field of cardiovascular diseases, usually occurs as a result of thrombosis or occlusion of a blood vessel, and it constitutes the most frequent complication and the leading cause of death in this category of diseases.^132^ After myocardial infarction, a significant increase in the cardiac expression of RBM15 has been observed. This elevation in RBM15 expression is closely associated with the methylation-mediated stabilization of NEDD8 activating enzyme E1 subunit 1 (NAE1) mRNA, a key mechanism that counteracts hypoxia-induced cell death and enhances cardiac function. The role of RBM15 in modulating NAE1 is further underscored by the marked increase in apoptotic markers, such as caspase-3 (CASP3) and Bcl-2-associated X protein (Bax), following the suppression of NAE1.^133^ Meanwhile, in lipopolysaccharide-treated myocardial injury models, RBM15 down-regulation indicates its potential role in the heart's defense against inflammation-induced damage.^134^

### RBM15 in metabolism

RBM15 exerts its influence on cellular nutrient uptake and utilization primarily by modulating the m^6^A methylation of various endogenous molecules. Within hepatic cells, RBM15 has been shown to govern the expression of genes integral to nutrient metabolism, particularly those involved in glucose and lipid homeostasis.^135^ This regulation is instrumental in shaping hepatic metabolic pathways, including gluconeogenesis and lipogenesis, which are critical for maintaining energy balance and cellular function.^136^ The precise mechanisms by which RBM15 orchestrates these processes suggest its potential as a metabolic regulator and a key factor in the liver's adaptive responses to nutritional changes.^137^^,^^138^ In the realm of glucose metabolism, RBM15 exhibits a strong correlation with the initiation and progression of insulin resistance. In a mouse model of gestational diabetes mellitus, m^6^A methylation of claudin-4 (CLDN4) by RBM15 reduces the efficiency of CLDN4 expression, resulting in reduced insulin sensitivity and insulin resistance in gestational diabetes mellitus offspring.^139^ Diabetic nephropathy emerges as a significant microvascular complication, intricately linked to metabolic dysregulations within the diabetic milieu.^140^ The role of RBM15 in diabetic nephropathy pathogenesis is increasingly recognized: it modulates the AGE–RAGE pathway through epigenetic mechanisms, influencing the expression of key proteins within this axis. Specifically, RBM15 has been shown to enhance the signaling cascade of the AGE–RAGE pathway, which in turn impedes the proliferation of the murine HK-2 cell line, a model of renal tubular cells.^141^ This inhibition of cellular proliferation is a result of exacerbating diabetic nephropathy by triggering a series of deleterious effects, including the promotion of inflammatory responses, induction of oxidative stress, and facilitation of cellular coagulation.^138^

In the context of lipid metabolism, RBM15 emerges as a crucial player in nonalcoholic fatty liver disease, a condition intimately linked to three pivotal detrimental elements: lipid accumulation, inflammation, and fibrosis.^142^ Studies have demonstrated that RBM15 contributes to enhancing lipid deposition and inflammation in nonalcoholic fatty liver disease, thereby influencing its progression.^137^^,^^143^ Notably, both RBM15 and WTAP exhibit reduced expression levels in nonalcoholic fatty liver disease. Li et al revealed an intriguing mechanism whereby RBM15 overexpression, facilitated by m^6^A methylation modification, up-regulates ring finger protein 5 (RNF5), which in turn down-regulates Rho-associated coiled-coil containing protein kinase 1 (ROCK1) protein, a crucial inhibitor of inflammatory cell migration.^143^ This down-regulation of ROCK1 mitigates inflammatory responses, ultimately improving the prognosis of nonalcoholic fatty liver disease patients. However, the precise relationship between RBM15 and fibrosis in nonalcoholic fatty liver disease remains elusive and necessitates further scientific exploration.

Abdominal aortic aneurysm is a life-threatening cardiovascular condition. The pathogenesis of this disease is significantly influenced by immune-mediated infiltration and the degradation of the aortic wall during the progression of abdominal aortic aneurysm.^144^ In abdominal aortic aneurysm, the elevated expression of RBM15 is implicated in disease progression, notably by enhancing apoptotic activities and modulating immune cell infiltration.^145^ RBM15 exerts its effects through the regulation of mRNA methylation, specifically by targeting CASP3, a pivotal enzyme in the execution of apoptosis.^146^ This interaction is crucial for modulating apoptotic and inflammatory responses within abdominal aortic aneurysm cells.^147^ Consequently, up-regulation of CASP3 intensifies apoptotic processes in human aortic smooth muscle cells and exhibits a positive correlation with the levels of macrophage infiltration in the adjacent tissue environment.^148^

**Figure 3:**
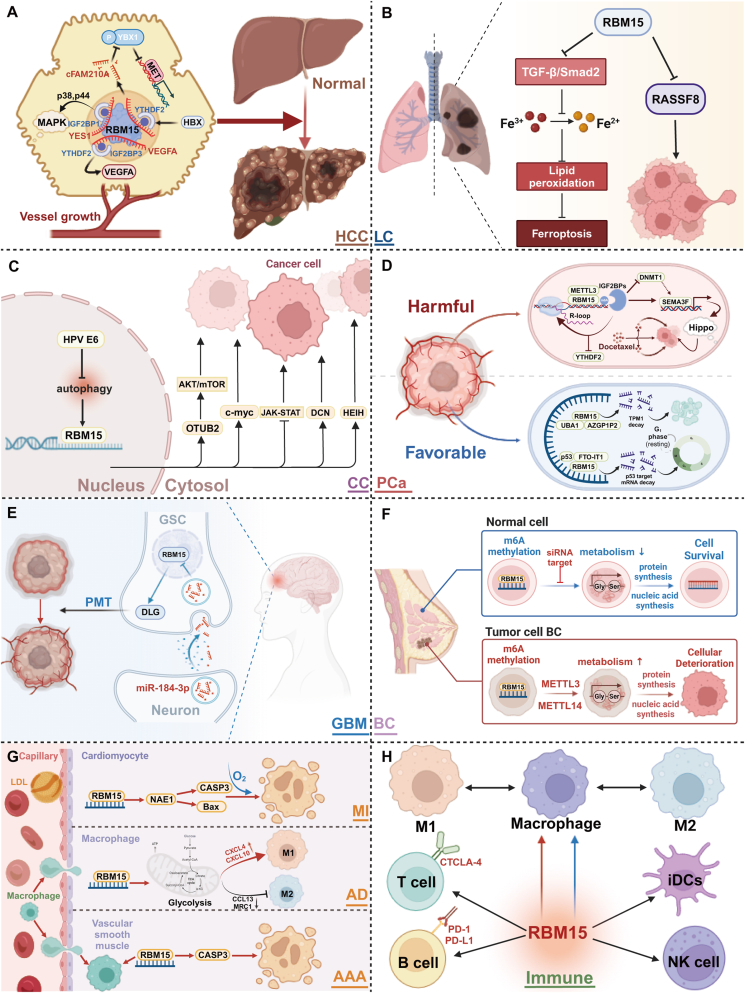
Specific mechanisms of RBM15 involvement in various diseases. **(A)** In the process of hepatocellular carcinoma, RBM15 triggers a series of complex biochemical reactions by promoting the methylation of mRNAs, such as YES1, VEGFA, and cFAM210A, and ultimately accelerates the process of malignant transformation in the liver. **(B)** In lung cancer, RBM15 inhibits iron death of cancer cells by suppressing the TGF-β/Smad2 pathway on the one hand and inhibits the protein level of RASSF8 on the other to promote cancer progression. **(C)** In cervical cancer, RBM15 is regulated by HPV E6 and actively promotes cancer progression by regulating downstream molecules or pathways, such as OTUB2, c-myc, JAK-STAT, DCN, and HEIH. **(D)** In prostate cancer, RBM15 plays a dual role: on the one hand, it interacts with p53 and FTO-IT1 to regulate the cell cycle or cooperates with UBA1 and AZGP1P2 to promote apoptosis; on the other hand, it enhances the resistance of the cells to docetaxel by mediating the R-loop methylation to be recognized by IGF2BPs. **(E)** In glioblastoma, miR-184-3p released by neurons is taken up by glioma stem cells and activates RBM15-mediated DLG methylation, contributing to the malignant transformation of normal cells. **(F)** In breast cancer, RBM15 provides favorable conditions for cancer development mainly by regulating the metabolic pathway of serine and glycine. **(G)** RBM15 also exacerbates the severity of myocardial infarction, aortic aneurysm, and entrapment, and abdominal aortic aneurysm by modifying NAE1, glycolysis-related molecules, and CASP3 through m^6^A methylation, respectively. **(H)** In the immune system, RBM15 plays a regulatory role and affects the infiltration status of immune cells, including macrophage polarization, activity, and function of T cells, B cells, dendritic cells, and natural killer cells.

In summary, RBM15 exerts a multifaceted role in the progression of diseases, potentially acting as either a promoter or an inhibitor, depending on the cellular context. These dualistic effects are a reflection of RBM15's intricate regulation of diverse cellular mechanisms, highlighting its adaptability and significance in disease pathology (Fig. 3).

### RBM15 in immunity and inflammation

Existing studies have shown that RBM15, as an m^6^A writer, is able to influence disease progression directly or indirectly by regulating immune-inflammatory cells (mainly macrophages).

On the one hand, RBM15 can directly promote disease progression by altering the immune function of macrophages. After overexpression of RBM15 in macrophages, increased levels of the M1-type macrophage markers chemokine (C-X-C motif) ligand 9 (CXCL9) and CXCL10 are observed.^149^ Within the realm of vascular pathologies, such as aortic aneurysms and aortic coarctation, RBM15 has been demonstrated to augment cellular energy metabolism within macrophages. This enhancement is mediated through the up-regulation of glycolytic pathways, which subsequently facilitates the polarization towards the M1 macrophage phenotype. Importantly, this metabolic shift is implicated in the acceleration of aortic coarctation's pathological progression.^150^

On the other hand, RBM15 influences tumor cell invasion and metastasis by interacting with the tumor immune microenvironment. A pan-cancer analysis has demonstrated a positive correlation between RBM15 gene expression and the extent of immune cell infiltration in clear cell renal cell carcinoma, lower-grade glioma, and pancreatic cancer. Notably, experimental evidence has firmly established that the suppression of RBM15 gene expression not only arrests the progression of pancreatic cancer but also stimulates macrophage infiltration and enhances the phagocytic activity of macrophages against pancreatic cancer cells.^151^ RBM15 was reported to promote macrophage infiltration and M2 polarization in renal cancer by promoting CXCL11 secretion in clear cell renal cell carcinoma cells *in vitro* and *in vivo*, and a high risk of RBM15 m^6^A in renal cancer can inhibit not only immunotherapy-related biological processes but also the expression of immune checkpoint markers.^113^^,^^152^

## RBM15 in clinical therapy

**Figure 4:**
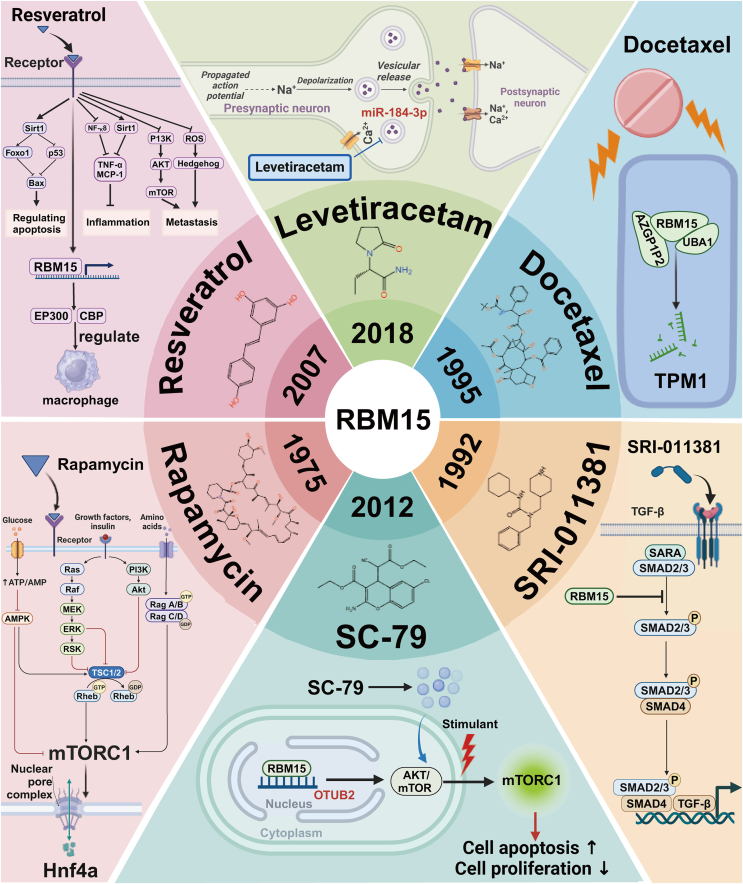
Agents related to RBM15 in clinical therapeutics. The core layer marks the initial point in time when each reagent was invented or discovered; the immediately following middle layer reveals the chemical structural features of the reagents and their proprietary names; and the most peripheral layer exhaustively elaborates on how these reagents interact with RBM15 at the cellular level through specific mechanisms, which in turn affect the disease process. Levetiracetam: In the treatment of gliomas, it indirectly blocks the function of RBM15 by inhibiting the uptake of miR-184-3p-rich exosomes by tumor cells, leading to therapeutic effects. Resveratrol: In the therapeutic strategy of clear cell renal cell carcinoma, resveratrol effectively inhibits cancer progression by activating the RBM15-mediated m^6^A methylation process and regulating the polarization status of macrophages. Docetaxel: In prostate cancer, RBM15 promotes the degradation of TPM1 mRNA, which in turn enhances the sensitivity of cancer cells to docetaxel, making the treatment more effective. SRI-011381: In the treatment of lung cancer, SRI-011381, as an activator of the TGF-β/Smad signaling pathway, by reversing the inhibitory effect of RBM15 on this signaling pathway, delays the progression of the disease. Rapamycin: As an activator of the mTOR signaling pathway, rapamycin partially restores the normal gene expression pattern in the liver by stimulating mTORC1 and promotes the nuclear translocation of the key transcription factor, Hnf4a, thus mitigating the detrimental effects of the RBM15 deletion. SC-79: In cervical cancer, SC-79 partially alleviates the inhibitory effect of rbm15-induced down-regulation of OTUB2 on the AKT/mTOR signaling pathway, and attenuates the malignant features of cancer cells.

Agents targeting the RBM15-related pathway are rapidly becoming a hotspot of research, and these agents show great potential and broad prospects in the treatment of a wide range of diseases, particularly in the field of oncology. Furthermore, a variety of agents, including levetiracetam, resveratrol, docetaxel, SRI-011381, rapamycin, and SC-79, have been recently identified as potential candidates for ameliorating cancer by targeting the signaling pathways associated with RBM15 (Fig. 4).^64^^,^^75^^,^^123^^,^^125^^,^^153^^,^^154^

Ma et al found that silencing RBM15 using RNA interference techniques enhanced myeloid differentiation of hematopoietic cells.^53^ However, no molecular inhibitors directly targeting RBM15 have been introduced into the clinic, mainly due to the complexity of RBM15 function and the relative lack of research in this area. On the one hand, RBM15 is extensively involved in the regulation of normal cellular physiological processes, and direct inhibition of RBM15 may increase the potential risk of serious side effects such as myelosuppression. In contrast, inhibition of RBM15 downstream molecules, such as IGF2BP3, which are often directly linked to specific pro-oncogenic pathways, significantly reduces the risk of global inhibition.^155^ In addition, as described above, RBM15 exhibits functional duality in certain diseases, which must also be considered. On the other hand, many key functions of RBM15, such as its synergistic regulation of the m^6^A methylation writing process with WTAP, were not gradually revealed until after 2017.^36^ Therefore, the large amount of research data supporting the development of RBM15 inhibitors is still insufficient at present, making it difficult to prioritize RBM15.

## Conclusion and perspectives

RBM15, a key factor in mRNA m^6^A modification, is crucial for cellular equilibrium and has a significant role in diseases, particularly in tumor initiation and progression. It regulates gene expression, which could aid therapeutic interventions and biomarker development. However, its effects vary across cancer types, possibly due to tumor heterogeneity or different molecular landscapes. This complexity highlights the need for further research to understand RBM15's precise mechanisms in cancer development.

Recent research has yielded several potential therapeutic strategies. In acute myeloid leukemia, the t(1; 22) translocation fuses RBM15 to the MKL1 gene to form an oncogenic fusion protein, RBM-MKL1.^156^ Knocking down the RBM15 gene can be used to block the formation of oncogenic fusion proteins using CRISPR-Cas9 gene editing. In addition to this, most studies have shown that the ubiquitination modification of RBM15 is aberrant in pathological states, especially when it is overexpressed in malignant tumors.^46^^,^^125^ Also, proteolysis-targeting chimera (PROTAC) molecules targeting the m^6^A methylation core complex METTL3–METTL14 have been successfully developed and shown anti-tumor activity.^157^ Therefore, PROTAC molecules targeting RBM15 can be designed in the future to specifically induce RBM15 protein degradation using the intracellular ubiquitin-proteasome system, thereby interfering with its abnormal function in disease progression.

Despite RBM15's potential as a clinical target, challenges remain. For instance, although studies have been carried out to reveal the synergistic effect of RBM15B on RBM15 at the cellular level, this has not been validated in specific mouse models. This is not difficult to understand because of the complexity of constructing a two-gene mouse model that affects both RBM15 and RBM15B; at the same time, most of the existing studies have focused on exploring the mechanism of action of either RBM15 or RBM15B alone in the disease, while there is a lack of studies on the combined effect of the two. In addition to WTAP, does RBM15 directly interact with other important writers such as METTL3/14? If so, further specific mechanisms of action need to be investigated. Also, RBM15 has a specific SPOC domain, which mainly regulates the m^6^A modification and increases mRNA stability, raising the question of the relevance of the SPOC domain to disease and its relationship to regulatory mechanisms, which is poorly understood.

In summary, current research findings highlight the critical role of the RBM15 gene in various diseases, laying the foundation for the development of novel therapeutic approaches. Future studies should focus on comprehensively elucidating the role of RBM15 in different (sub)types of diseases and its molecular mechanisms, advancing the development of innovative therapies such as small-molecule inhibitors directly targeting RBM15 and indirect intervention strategies (*e.g.*, PROTAC degraders). Concurrently, preclinical validation should be strengthened, including the establishment of patient-derived xenograft models and disease organoids, to optimize treatment strategies and assess potential side effects. Additionally, based on RBM15-related biomarkers and patient molecular subtyping, prospective design of phase I/II clinical trials should be conducted to validate efficacy and safety in clearly defined patient subgroups, while concurrently identifying predictive biomarkers to advance the clinical translation of RBM15-targeted therapies.

## CRediT authorship contribution statement

**Fengze Li:** Writing – original draft. **Junzhe Liu:** Writing – original draft. **Na Wang:** Writing – original draft. **Zhihong Zhou:** Writing – review & editing. **Linzhen Huang:** Writing – review & editing. **Qiankun Ji:** Supervision. **Jingying Li:** Supervision, Funding acquisition.

## Funding

This work was supported by the 10.13039/501100001809National Natural Science Foundation of China (No. 82260524, 82460503 to Jingying Li) and Jiangxi Province Department of Education Science and Technology Research Project (China) (No. GJJ210177 to Jingying Li).

## Conflict of interests

The authors declared no competing interests.
